# Supplementary material for: Beliefs and Perceptions in Attending the Cervical Screening: The COMUNISS Project Experience
Source: Cancers (Basel). 2025 Jan 9;17(2):190. doi: 10.3390/cancers17020190 (PMC11763979; doi:10.3390/cancers17020190)
Supplement: Supplementary file 1 [file cancers-17-00190-s001.zip › cancers-3393855-supplementary.pdf]

Supplementary materials_S1:

Format of questionnaire

1. **Socio-demographic and clinical data**
    1. Municipality of residence
    2. Age
    3. Civil status
    4. Employment status
    5. Level of education
    6. Have you ever had a Pap test in the past?

2. **Health Belief Models Questionnaire (Likert Scale responses: Disagree, Neutral, Agree)**

Perceived Susceptibility	- There is a very low probability that I will develop cervical cancer lesions<br>- I may be at risk of having contracted HPV infection in the past<br>- In the absence of symptoms, I do not have to worry about the risk of having developed cervical lesions
Perceived Severity	- HPV lesions are a serious health problem<br>- If I were to test positive for HPV, my health would be seriously at risk.<br>- Cervical cancer would put my health at serious risk.<br>- Treatments for cervical cancer are not very effective.
Perceived Benefits	- Screening ensures early diagnosis and successful treatment<br>- Having a Pap test every 3 years reduces the risk of developing cervical cancer.
Perceived Barriers	- Having a gynecological examination creates fear and anxiety for me.<br>- Having a Pap test is a painful and embarrassing procedure
Cues to action	- Participating in screening is a priority for me<br>- Protecting my health is a priority for me
Self-efficacy	- Making an appointment, even with my busy schedule, is possible.<br>- I can find out through my general practitioner how to get an appointment at the screening center.

3. **Test of Knowledge items (True/false answers):**

1. Cervical screening reduces the risk of developing malignant lesions.
2. HPV infection is the main cause of cervical cancer.
3. HPV infection can also cause malignant lesions in the anus and oro-pharynx.
4. Vaccination is an effective tool for preventing HPV infection.
5. HPV infection only affects women.
6. I can have the vaccination even if I am over 25 years old.
7. After vaccination, it is not necessary to participate in screening.
8. The vaccination is also offered to adolescent males.
9. The HPV infection is mainly transmitted sexually.
10. It is possible to undergo cervical screening by taking a vaginal swab, collected independently, without going to the screening center.
11. The self-collected vaginal swab is a reliable alternative for HPV screening.

Table S1: Demographic and clinical data

Socio-demographic and clinical data	N	%
**Age group**		
19-28	63	25,1%
29-38	90	35,9%
39-48	53	21,1%
49-59	39	15,5%
60-75	6	2,4%
**Civil status**		
Single	108	43%
Married	140	56%
Widow	1	1%
**Level of Education**		
Degree	112	44,4%
High School	121	48,0%
Middle School	19	7,5%
**Occupational status**		
Employee	35	13,9%
Unemployed	154	61,1%
Student	63	25,0%

**Have you ever had a Pap test in the past?**		
Yes, regularly	130	51,6%
Never/Not regularly	122	48,4%

Table S2: Differences in screening uptake by socio-demographic variables

Socio-demographic variables			
**Age group**	*Yes, regularly, N (%)*	*Never/Not regulalry , N (%)*	*P-value*
19-28	3.9	47.9	
29-38	46.9	24.0	
39-48	25.4	16.5	<0.001
49-59	20.8	9.9	
60-75	3.4	1.7	
**Civil status**	*Yes, regularly, N (%)*	*Never/Not regulalry , N (%)*	
Single	31 (42.5)	77 (44.0)	0.82
Married	42 (57.5)	98 (56.0)	
**Level of Education**	*Yes, regularly, N (%)*	*Never/Not regulalry , N (%)*	
Degree	72 (55.4)	40 (33.1)	
High School	48 (37.0)	72 (60.0)	<0.001
Middle School	10 (7.7)	9 (7.4)	
**Occupational status**	*Yes, regularly, N (%)*	*Never/Not regulalry , N (%)*	

Employee	104 (80.6)	49 (40.2)	
Unemployed	15 (11.6)	20 (16.4)	<0.001
Student	10 (7.8)	53 (43.4)	

Table S3: Median score of Health Belief Model test among participants, using a Rickert scale range: "disagree", "neutral", and "agree", with a corresponding score of 1, 2 and 3, respectively.

HBM items	Sentence	Median (IQR)
Perceived Susceptibility	There is a very low probability that I will develop cervical cancer lesions	2(1-2)
	I may be at risk of having contracted HPV infection in the past	2(1-3)
	In the absence of symptoms, I do not have to worry about the risk of having developed cervical lesions	1(1-1)
Perceived Severity	HPV lesions are a serious health problem	3(3-3)
	If I were to test positive for HPV, my health would be seriously at risk.	3(2-3)
	Cervical cancer would put my health at serious risk.	3(3-3)
	Treatments for cervical cancer are not very effective.	1(1-2)
Perceived Benefits	Screening ensures early diagnosis and successful treatment	3(3-3)
	Having a Pap test every 3 years reduces the risk of developing cervical cancer.	3(3-3)
Perceived Barriers	Having a gynecological examination creates fear and anxiety for me.	1(1-2)
	Having a Pap test is a painful and embarrassing procedure	1(1-2)
Cues to action	Participating in screening is a priority for me	3(3-3)
	Protecting my health is a priority for me	3(3-3)
Self-efficacy	Making an appointment, even with my busy schedule, is possible.	2(2-3)
	I can find out through my general practitioner how to get an appointment at the screening center.	1(1-3)

Table S4: Results of test of knowledge by true/false modality.

Test of knowledge (True/False)	Correct (%)	Incorrect (%)
Cervical screening reduces the risk of developing malignant lesions.	90%	10%
HPV infection is the main cause of cervical cancer.	89%	11%
HPV infection can also cause malignant lesions in the anus and oro-pharynx.	83%	17%
Vaccination is an effective tool for preventing HPV infection.	93%	7%
HPV infection only affects women.	84%	16%
I can have the vaccination even if I am over 25 years old.	82%	18%
After vaccination, it is not necessary to participate in screening.	96%	4%
The vaccination is also offered to adolescent males.	78%	22%
The HPV infection is mainly transmitted sexually.	80%	20%
It is possible to undergo cervical screening by taking a vaginal swab, collected independently, without going to the screening center.	78%	22%

The self-collected vaginal swab is a reliable alternative for HPV screening.	79%	21%
